# The Management of Distal Clavicle Fractures – A Survey of UK Shoulder and Elbow Surgeons

**DOI:** 10.7759/cureus.17305

**Published:** 2021-08-19

**Authors:** Vivek Sharma, Amit Modi, Alison Armstrong, Radhakant Pandey, Dhiraj Sharma, Harvinder Singh

**Affiliations:** 1 Department of Trauma and Orthopaedics, Leicester Royal Infirmary, Leicester, GBR; 2 Department of Trauma and Orthopaedics, Norfolk and Norwich University Hospital, Norwich, GBR

**Keywords:** distal clavicle, fracture, orthopeadics, shoulder, upper limb surgery, survery

## Abstract

Background

Distal clavicle fractures (DCF) are a management challenge frequently encountered by shoulder surgeons. Despite an array of surgical fixation strategies, the indications and role of surgery are unclear, with there being no gold standard or consensus regarding their management. The aim of this study was to identify current United Kingdom (UK) clinical practices relating to DCFs and to inform a future randomised control trial (RCT).

Methods

An online survey was sent to the consultant surgeon members of the British Elbow and Shoulder Society (BESS). Questions covered respondent indications for surgical fixation, important factors considered for management of DCFs, fixation strategies, the volume of patients treated, and willingness to participate in the conduct of a randomized trial.

Results

The response rate was 84/327 (26%). 64-67% of respondents reported surgically managing DCFs classified as Neer type 2A, 2B and 5. The most important factors considered by surgeons when deciding between operative and nonoperative intervention were degree of displacement (90%), clinical assessment of impending open fracture (87%), and age of the patient (74%). For conservatively managed DCFs, the preferred length of complete immobilization was 2-4 weeks (46%), followed by 4-8 weeks (17%). 30% reported not immobilizing their patients at all. For operative intervention, the locking plate was the preferred fixation method by most respondents (68%), although there was no clear consensus regarding other fixation methods. Most surgeons (52%) reported treating a low volume of patients with DCFs (0-10) per year. 58% of respondents were willing to randomize patients to non-operative treatment in a multi-centre RCT, with a further 22% undecided. Finally, 68% (n=79) of respondents would consider being co-investigators in such a trial.

Conclusion

There is considerable heterogeneity in the management of patients with DCFs in the UK. The indications for surgery and the optimal surgical fixation method remain uncertain. There is a clear need for pragmatic multi-centre clinical research in this area.

## Introduction

Fractures of the clavicle account for 2-4% of all fractures, of which injuries affecting the distal clavicle third account for 10-30%. They show a bimodal distribution of occurrence between 15 and 24 years, usually following sports injuries, and over 65 years, usually following falls [1]. Although relatively uncommon, distal clavicle fractures (DCF) are a management challenge frequently encountered by shoulder surgeons. Despite an arsenal of surgical treatment options available, there is currently no established gold standard or consensus regarding their management.

The challenge of DCFs arises from the inherent instability of certain fracture patterns, which can be difficult to discern radiographically [2]. Non-union rates following nonoperative management range in the literature from 11% to 40% [3]. Therefore, surgical treatment is usually recommended for unstable Neer type II and V clavicle fractures [3,4]. However, the clinical significance of non-union is uncertain, with many patient’s remaining asymptomatic [5]. Combining this with the significant reported complications and re-operation rates of operative fixation, as well as the poorly defined indications, the role of operative fixation is unclear [5-7].

To our knowledge, there is only one registered multi-centre randomized control trial (RCT) comparing nonoperative treatment to open reduction internal fixation (ORIF) with a plate for distal clavicle fractures. In total, only 57 patients were recruited to the trial - 30 in the operative arm and 27 in the conservatively managed arm. Although patients treated operatively had higher rates of union (96% vs 63%, P = 0.01), there was no difference in patient-reported outcome scores (disabilities of the arm, shoulder and hand (DASH) and constant) after one year [8].

The aim of this survey was to identify current United Kingdom (UK) clinical practices relating to displaced DCFs to inform a multi-centre RCT. We were particularly interested in the proportion of patients that are treated surgically and what factors influence surgical decision making. These questions are increasingly important as the incidence and rate of surgery for DCFs continues to rise with an ageing population [9,10]. In the backdrop of a scarcity of clinical evidence, understanding divergences in clinical practice are essential for designing and implementing pragmatic multi-centre clinical trials.

## Materials and methods

Survey contents

A data collection questionnaire was developed to investigate the factors affecting variations in decision making of UK surgeons when treating patients with DCFs. We first sought to understand which fracture characteristics and patient factors contribute to surgical decision making. To determine standard non-operative management, we questioned the length of time clinicians preferred to immobilize patients. We then ascertained how many patients with DCFs the surgeon treated per annum, the proportion of which was managed surgically, and their operative fixation method of choice. Finally, as the purpose of the questionnaire was to inform the development of a future multicentre trial, we attempted to identify surgeons willing to participate or become co-investigator in such a study.

Administration of survey

The survey was initially trialled within the department with four surgeon members of the upper limb team before submission for consideration to the British Elbow and Shoulder Society (BESS) scientific committee. Once approved, all surgeon members of the BESS (consultant and senior speciality trainees) were invited to participate in an online survey (Appendix) using the Qualtrics software (Qualtrics, Provo, Utah). In total, the survey consisted of 9 questions to gain as much meaningful information from individual surgeons as possible without burdening them. No minimum number of responses were required, and the response rate was calculated based on the number of responses and the BESS membership at the time. Duplicate answers were excluded automatically using the Qualtrics software. Qualtrics software tracks responses by IP address and the location of the device used for the response, preventing multiple responses from the same IP address or location. Descriptive analysis was used to summarize the findings of the survey. Microsoft Excel 2020 (Microsoft Corporation, Redmond, Washington) was used for graphical illustrations. The invitation email was first distributed on 30th April 2019, and following reminders, the survey closed on 21st June 2019. The survey was deemed to be a service improvement exercise; therefore, the project did not require multi-site ethical, research governance, or audit approval.

A copy of the survey can be found in the appendix (Figure 5-7).

## Results

Indications for surgical treatment

**Figure 1:**
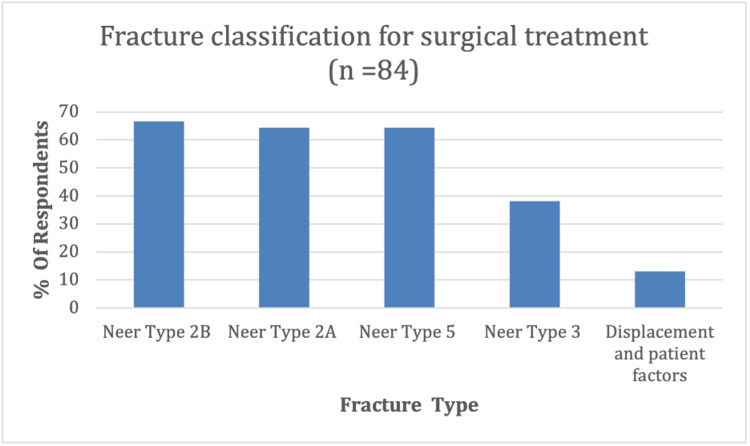
DCFs managed surgically categorized by Neer’s classification DCF: distal clavicle fracture

A total of 84 (26%) responses were received from 327 (2018-2019) surgeon members of BESS (consultant and senior speciality trainees). No demographic data about the respondents were collected, apart from voluntary details about their National Health Service (NHS) trust and contact address. A number of questions allowed multiple answers, and therefore the number of responses for some questions were higher than 84. The first question received a total of 207 responses, determining which Neer fracture classification of DCFs the respondent would manage surgically. The preferred fracture configuration for surgical management by respondents was Neer type 2A,2B and five, selected by 64-67% of surgeons (n=84) (Figure 1).

38% reported surgically managing Neer type three fractures, and 13% (responding in the ‘others’ section) reported not using Neer’s fracture classification to determine operative management; instead, using a degree of displacement and patient factors as more important indicators.

The second question asked respondents which factors were most important when deciding between operative and non-operative management. The most important factors considered by our respondents were degree of displacement (90%, n=84)(Table 1), clinical assessment of impending open fracture (87%), age of the patient (74%), and fracture comminution (44%). With over 273 responses garnered, other important factors included patient co-morbidities (12%), patient choice (5%), activity status (4%).

**Table 1:** Factors considered important when deciding between operative and non-operative management of the distal clavicle.

Factors considered	Number of respondents: n (% = n/84)
Displacement of the fracture	76 (90)
Clinical assessment (skin puckering/tenting)	73 (87)
Age of the patient	62 (74)
Comminution of the fracture	37 (44)
Co-morbidities e.g. diabetes,	10 (12)
Smoking	6 (7)
Patient choice	4 (5)
Activity status	3 (4)
Others	2 (2)

The management of DCFs

**Figure 2:**
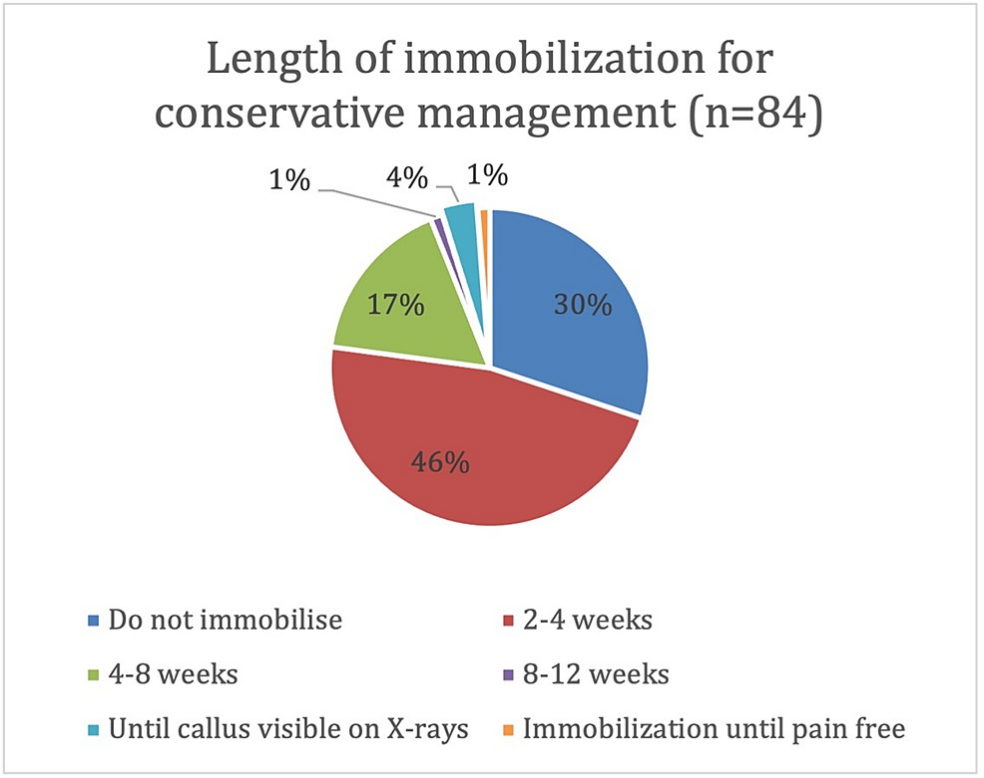
Length of time DCFs managed conservatively are completely immobilised for DCF: distal clavicle fractures

The next question dealt with how the respondents conservatively managed DCFs. The preferred length of complete immobilization was 2-4 weeks (46%, n =84) (Figure 2), followed by 4-8 weeks (17%). 30% of respondents reported not immobilizing their patients at all. 

The most important factor when treating displaced DCFs was whether they were open or not (93%, n=84) (Table 2). This was followed by patient choice (78%), age of the patient (75%), associated nerve or vessel injury (70%), and multiple long bone fractures (69%). Other factors considered important by respondents included pathological fractures (51%), smoking (40%) and diabetes (20%). 420 responses were received for this question. 

**Table 2:** Factors considered important when deciding between operative and non-operative management of the distal clavicle

Factors considered	Number of respondents: n (% = n/84)
Open fractures	77 (93)
Patient choice	65 (78)
Age of patient	62 (75)
Associated nerve or vessel injury	58 (70)
Multiple long bone fractures	57 (69)
Pathological fractures	42 (51)
Smoking	33 (40)
Diabetes	23 (28)
Others	3 (4)

**Figure 3:**
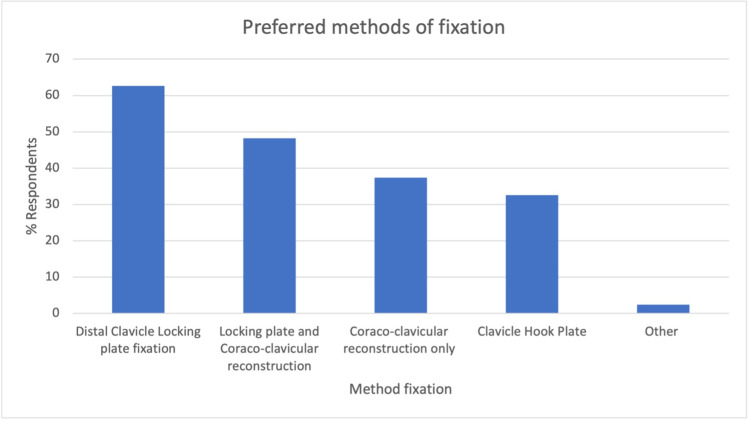
Respondents preferred methods of DCF fixation DCF: distal clavicle fracture

The next question determined the fixation method of choice by respondents for surgically managed patients. Of the 152 responses (n=84), the majority of respondents (63%) (Figure 3) chose the distal clavicle locking plate (LP). Once again, there was no clear consensus, with LP and coracoclavicular (CC) reconstruction (48%), CC reconstruction only (37%), and clavicle hook plate (HP) (33%) also commonly selected methods of fixation. A free-text box collected 11 additional responses, which included: transacromial fixation, tension band wiring, and acromioclavicular joint reconstruction.

Future Trials

The survey also attempted to estimate the recruitment rate from a multicentre national trial to assess for study feasibility. We asked respondents how many patients with DCFs they manage each year, along with what proportion they treated surgically. Most respondents (52%, n=84), treated 0- 10 patients each year, followed by 32% treating 10-20 patients, and 11% treating 20-35 patients (Table 3).

**Table 3:** Number of patients with DCFs managed in a year DCF: distal clavicle fracture

Number of patients with DCFs treated per year	Number of respondents: n (% = n/84)
0-10	44 (52)
10-20	27 (32)
20-35	9 (11)
35-50	2(2)
More than 50	2(2)

The proportion of such patients surgically managed varied widely between respondents, with an almost even distribution between most categories (Table 4).

**Table 4:** Proportion of patients with displaced DCFs managed primarily with surgery DCF: distal clavicle fracture

Proportion of displaced DCFs treated surgically	Number of respondents: n (% = n/81)
0-10%	6 (7)
10-25%	19 (23)
25-50%	23 (27)
50-75%	22 (26)
75-100%	14 (17)

Finally, there was significant interest in taking part in a national RCT. 58% of respondents felt that they had the equipoise and would be willing to randomize patients with displaced distal clavicle fractures to non-operative treatment in a multi-centre RCT, with a further 22% ‘maybe’ willing to consider this (n=81). Moreover, 68% of respondents would consider being co-investigators, with 48 individuals also providing their contact details for further information (n=79). 

## Discussion

We carried out a national survey of surgeon members of BESS to assess current management strategies for DCFs. The aim of early surgical intervention is to reduce the risk of future symptomatic non-union and the necessity for further operative procedures. Management is controversial, with surgical decisions primarily driven by the degree of fracture stability and displacement.

**Figure 4:**
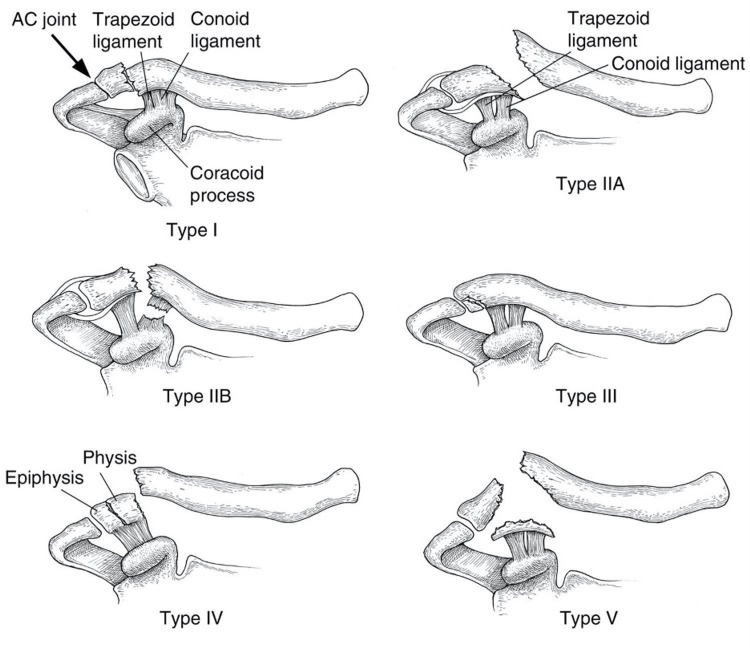
Modified Neer classification of DCFs DCF: distal clavicle fracture Contributed by Leah Ahn and Evan Watts via Wikimedia Commons.

**Figure 5:**
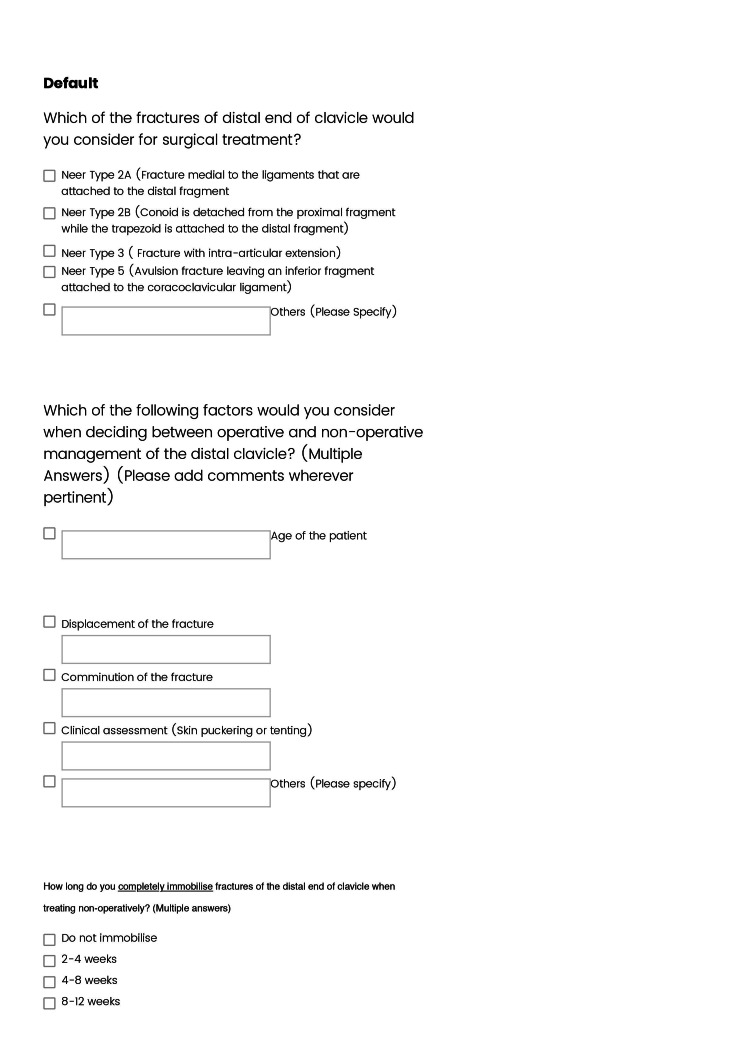
Online survey on DCFs (page 1) DCF: distal clavicle fracture

**Figure 6:**
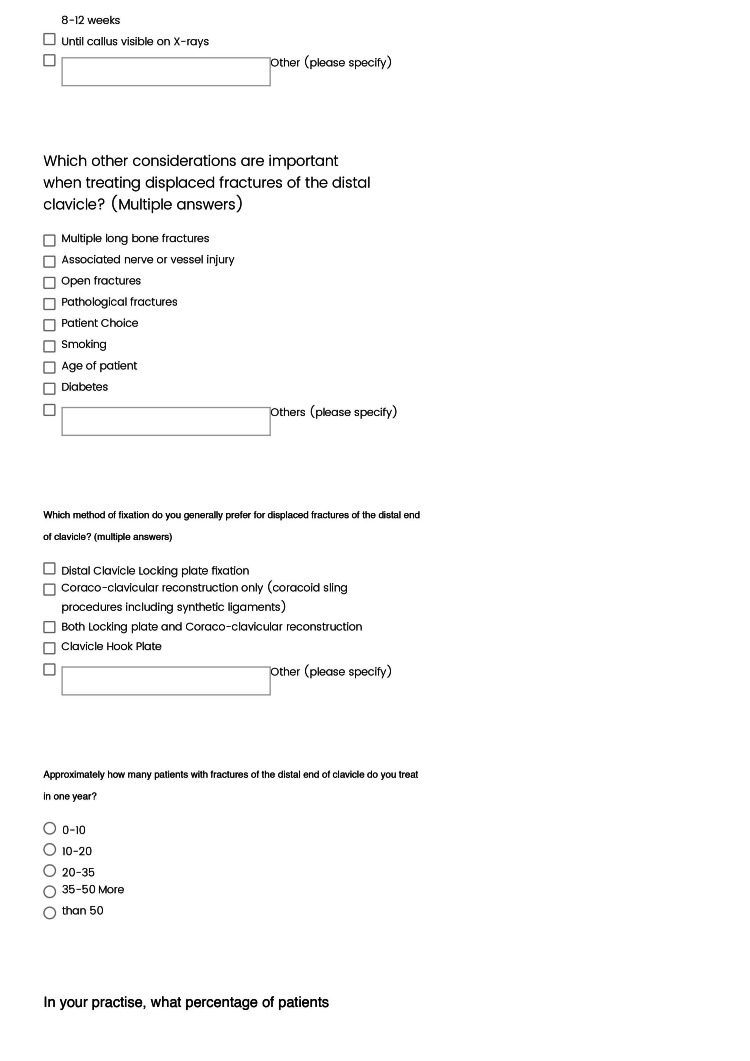
Online survey on DCFs (page 2) DCF: distal clavicle fracture

**Figure 7:**
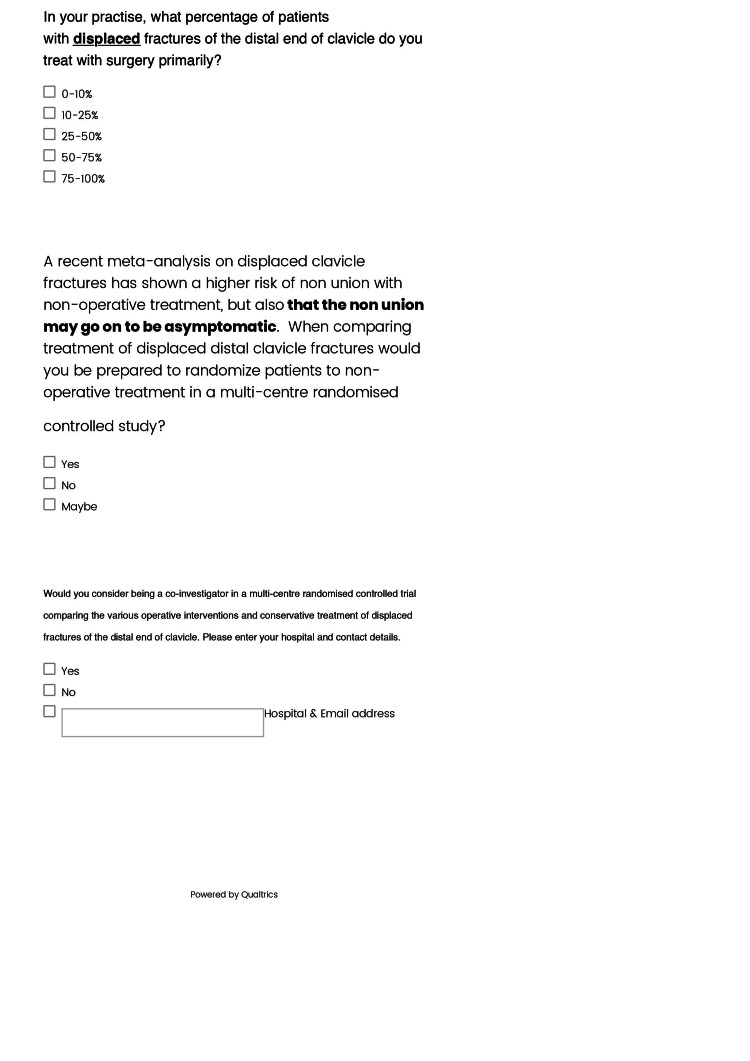
Online survey on DCFs (page 3) DCF: distal clavicle fracture

The modified Neer classification is the most widely reported classification system determining stability, first described by Neer in the 1960s and based on fracture location in relation to the CC ligament (Figure 4).

Types one and three are classified as stable and are usually managed conservatively. However, type three extends into the acromioclavicular (AC) joint, increasing the risk of traumatic AC joint arthritis. This may explain why 38% of respondents surgically manage these fractures [11]. Nevertheless, most respondents (64-67%) reported surgically managing Neer type 2A, 2B and type five fractures. These fracture configurations are considered unstable and have historically been managed surgically due to the increased risk of non-union and associated functional limitations if managed conservatively [3]. However, only a fair level of inter-rater agreement has been reported for the modified Neer classification, with many fracture configurations not fitting into discrete classes [2,12]. Therefore, its clinical utility is unclear. For these reasons, several authors have developed new classification systems. A comparison of DCF classification systems by O'Neill et al. showed Craig’s classification, which expands on Neer’s by differentiating between ligamentous injury patterns, intra-articular, and paediatric fractures, was most prognostic when predicting delayed union or nonunion [13]. However, Craig’s classification is yet to be validated. More recently, Cho et al. developed a new classification system considering fracture displacement, stability, as well as location. This classification system showed moderate interobserver and substantial intra-observer reliability but has yet to be widely adopted [5]. These controversies may explain why 13% of respondents did not use the Neer classification instead of making surgical decisions on the degree of displacement and patient factors.

Almost unanimously, the most important factors driving operative management reported by respondents were fracture displacement (90%) and clinical features of an impending open fracture (87%). The next most important factors were the age of the patient (75%) and fracture comminution (44%). Such factors have been shown to increase the risk of non-union and have traditionally been considered unacceptable for conservative management, particularly in young patients with high functional demands and work commitments [14].

Most non-displaced fractures are managed conservatively with a sling. Such fractures typically heal without sequelae, although Neer type three fractures are theoretically at increased risk of traumatic arthrosis [5]. However, there appears to be no consensus on the optimal duration of immobilization. This is reflected in our survey, with 30% of respondents reporting not immobilizing patients at all, whilst 46% immobilized for 2-4 weeks and 17% for 4-8 weeks.

The management of displaced DCFs remains more controversial. Cairn’s et al. reported mid-term functional results of 101 patients with displaced DCFs treated conservatively of which, 14% developed symptomatic non-union requiring delayed surgical intervention and 21% developed asymptomatic non-union [15]. Importantly, there was no functional difference (as measured by Constant and Short Form-36 scores) between the patients with non-union and those in whom the fracture had healed, or comparing patients treated non-operatively and those who had delayed surgery [15]. Therefore, the functional impact of non-union is unclear. Rokito et al. compared the surgical and nonoperative treatments in a case series of 16 patients with Neer type two fractures. Once again, non-union had no significant effect on functional outcome or strength (Constant or American Shoulder and Elbow Surgeons scores) [7]. These findings are supported by Hall et al., who performed the only RCT reported in the literature. The authors compared ORIF with a plate to nonoperative treatment with a sling for completely displaced DCFs. Although the non-operatively managed group had higher rates of non-union, there were no differences between patient-reported outcome measures (DASH and Constant), return to work, or return to activity. Moreover, 12 patients in the operative group underwent a second operation for hardware removal (12/27, 44%), whilst only 6 patients in the nonoperative group underwent an operative procedure (6/30, 20%) [8].

Absolute indications for surgical intervention include open fractures (or impending open fractures) and associated vascular injury. Although controversial, most surgeons advocate surgical intervention for unstable and displaced fractures to prevent non-union and the traditionally presumed clinical sequelae, particularly in active young patients. Other important factors reported by our respondents included additional risk factors for non-union (Table 2). The patient choice was considered important by 78% of respondents. When a variety of treatments are available and no strategy is clearly optimal, shared decision making has been shown to improve patient satisfaction and adherence to therapy [16].

Various methods of surgical fixation of DCFs have been proposed, including K wire (KW) fixation, LP fixation with or without CC reconstruction, HP fixation, CC reconstruction only, tension band wiring (TB), and arthroscopic techniques. The results from our survey reflect the diversity of the literature, with most respondents preferring fixation with an LP (63%), followed by LP and CC reconstruction (48%), CC reconstruction only (37%), and HP fixation (33%). A recent meta-analysis, including 18 studies, found no significant difference in functional outcomes between fixation methods at three and six months and no difference in time to radiographic union. However, at more than one year, functional scores were significantly worse with KW compared to other fixation methods. For both the risk of re-operation and symptomatic implants, CC suturing with LP was associated with significantly lower risks than with all other interventions, although there was insufficient data to compare LP alone [17]. These findings are corroborated by Boonard et al., who performed a network meta-analysis on 11 studies, concluding CC fixation and LP fixation was functionally better than both the HP and TB for function and that LP was associated with the lowest risk of complications [18]. Despite high rates of union, the use the HP is particularly contentious due to high rates of irritation requiring hardware removal reported in the literature (62.5%) [6]. This has led to some authors routinely removing HP metalwork at four-six months to reduce the risk of postoperative complications [19].

Significant limitations to both analyses are the inclusion of poor quality, mainly observational studies, with low sample sizes. A contributing factor may also be the low volume of DCFs managed by single centres. In our survey, most respondents treated less than 10 patients with DCFs per year. Combining this with the variations of fixation methods across the UK, the need for collaboration between centres in the development of a large-scale prospective RCT is clear.

The main limitation of this survey is the low response rate of around 26% from the BESS membership; therefore, the findings may not reflect current clinical practice in the UK. However, the response rate is not dissimilar to other survey-based studies in the literature and given that our survey was directed more towards surgeon members, and a proportion of BESS members are other health care professionals or inactive, thus non-contactable, our response rate is likely to be much higher than that reported [20]. It is also important to consider the respondents in the context that this survey was designed to inform a national clinical trial; therefore, we may have attracted responses from participants who are generally more research-oriented. More research-orientated members are also more likely to complete surveys in the first place, and the responses may not be representative of the wider upper-limb surgical community. Responses to surveys may also not be an accurate reflection of the participant's actual practice at their institution, also being subject to reporting bias based on their clinical experience over the preceding few months. Despite these limitations, the survey was completed by consultants working in at least 48 different institutions within the UK (not all respondents provided institution details), and we believe our results provide a meaningful reflection of the diverse clinical practice. Finally, this study surveyed only UK surgeons with BESS membership. But results are likely to be generalizable to institutions worldwide; with no international consensus or gold standard recommendations, distal clavicle fractures represent a challenge to orthopaedic surgeons globally.

## Conclusions

There is considerable heterogeneity in the management of patients with DCFs in the UK. There appears to be no national consensus on the indications for surgical management or which fixation method to utilize. The results of this survey hold strong testimony for the need for a pragmatic multi-centre RCT comparing conservative and surgical management for displaced DCFs.
